# Atlantic Salmon Reovirus Infection Causes a CD8 T Cell Myocarditis in Atlantic Salmon (*Salmo salar* L.)

**DOI:** 10.1371/journal.pone.0037269

**Published:** 2012-06-05

**Authors:** Aase B. Mikalsen, Oyvind Haugland, Marit Rode, Inge Tom Solbakk, Oystein Evensen

**Affiliations:** 1 Department of Basic Sciences and Aquatic Medicine, Norwegian School of Veterinary Science, Oslo, Norway; 2 PHARMAQ AS, Oslo, Norway; INRA, France

## Abstract

Heart and skeletal inflammation (HSMI) of farmed Atlantic salmon (*Salmo salar* L.) is a disease characterized by a chronic myocarditis involving the epicardium and the compact and spongious part of the heart ventricle. Chronic myositis of the red skeletal muscle is also a typical finding of HSMI. Piscine reovirus (PRV) has been detected by real-time PCR from farmed and wild salmon with and without typical changes of HSMI and thus the causal relationship between presence of virus and the disease has not been fully determined [1]. In this study we show that the Atlantic salmon reovirus (ASRV), identical to PRV, can be passaged in GF-1 cells and experimental challenge of naïve Atlantic salmon with cell culture passaged reovirus results in cardiac and skeletal muscle pathology typical of HSMI with onset of pathology from 6 weeks, peaking by 9 weeks post challenge. ASRV replicates in heart tissue and the peak level of virus replication coincides with peak of heart lesions. We further demonstrate mRNA transcript assessment and in situ characterization that challenged fish develop a CD8^+^ T cell myocarditis.

## Introduction

Heart and skeletal muscle inflammation (HSMI) of farmed Atlantic salmon (*Salmo salar* L) has been known as a disease entity in seawater for more than 10 years [2]. The mortality in affected pens varies from almost insignificant up to 20%, while morbidity is close to 100% [2], [3]. Fish are found without obvious external signs, while internal gross changes are typical of circulatory disturbance. Most fish have a pale heart, yellowish liver, ascites, swollen spleen and petechiae in the perivisceral fat. The histopathological changes are seen primarily in heart and skeletal muscle which gave the name of the disease, and cardiac changes are diffuse infiltration of lymphocytic cells and myocardial degeneration in spongy and compact layers of the ventricle [2], [3]. The phenotype of the infiltrating cells has not been decided. HSMI has been considered having an infectious nature [2] and recently a reovirus, termed piscine reovirus (PRV), was detected from fish with clinical signs of HSMI. Occurrence of PRV in internal organs correlated with disease phase in field material from outbreaks of HSMI and PRV was found in fish infected experimentally with a HSMI-related tissue extract and their cohabitants [1]. Still, the virus was also found at low concentrations and low prevalence in both farmed and wild fish with no clinical signs of disease and the virus was not grown in cell cultures [1].

HSMI challenge studies have shown a time lag of 6–8 weeks from time of injection till the first signs of a myocarditis are seen [2]. Since it has not been possible to estimate the virus load in affected organs this raises a question as to whether the virus replicates in the heart and if there is any correlation between inflammatory changes in the heart and virus replication. T-cell immunity of fish in general and salmonids in particular has been subject of studies over the last years. CD8 and CD4 co-receptors have been cloned in several fish species, including salmonids [4]–[11] and recently it has been suggested that CD8α is a marker for distinct compartments of teleost T cells and NK cells [12]. Immune cells of salmonids carry membrane-bound major histocompatibility complex (MHC) class I and II molecules [13]–[15], indicative of classical restriction of T cell responses. The biological activities, like cytotoxic activities, of T-cell subpopulations have been studied [16] and various salmonid cytokines have been cloned and sequenced. Functional traits of IFNγ as an example suggest that the Th1 immune response is likely present in lower vertebrates [17], [18]. However, reagents detecting surface markers of teleost T-cells are scant which make functional studies difficult. Markers of salmon T cells (CD3ε) have been described [19], as has a monoclonal antibody specific for CD8 T cells of Atlantic salmon [20] together with antibodies against CD8α in rainbow trout (*Oncorhynchus mykiss*), ginbuna crucian carp (*Carassius auratus langsdorfli*) and fugu (*Takifugu rubripes*) [4], [12], [21] and CD4 in ginbuna crucian carp and green spotted puffer (*Tetraodeaon*) [21], [22].

In this study we show that virus genome sequences related to Reoviridae were identified in a cell culture inoculated with a HSMI-related tissue homogenate and the virus was termed Atlantic salmon reovirus (ASRV). The genome sequences are identical or nearly identical to PRV. Experimental challenge of naïve Atlantic salmon with ASRV passaged through cell culture resulted in cardiac and skeletal muscle pathology typical of HSMI. ASRV replicated in heart tissue and replication coincided with expression of the antiviral Mx protein. Virus load, measured by real-time PCR, correlated with development of histological heart lesions. We further demonstrate that the cells infiltrating the heart tissue was mainly CD8^+^ T cells and the virus replication peaked a few days prior to the inflammatory response. These findings show that HSMI is correlated with reovirus infection in an experimental challenge model, and is characterized as a T cell myocarditis.

## Results

### Cell Culture Passage and Physio-chemical Characterization of the Virus

**Figure 1:**
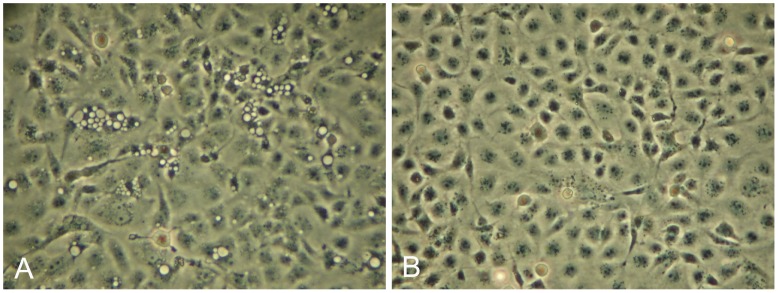
CPE in GF-1 cells. GF-1 cell cultures infected with sterile filtered heart tissue homogenate supernatant collected from a) a clinical outbreak of HSMI and b) from non-related healthy salmon (controls).

In cell cultures inoculated with filtered homogenate collected from fish with typical symptoms of HSMI and confirmed by histopathological examination, we observed cytoplasmic vacuoles approximately 1 week post inoculation (wpi). By two weeks, large vacuoles were observed throughout the monolayer (Fig. 1a). Transfer of supernatant to fresh uninfected cell cultures resulted in similar vacuolization and this was repeated up to 4^th^ passage, resulting in reduction of vacuolization with increasing passage number. Cytopathic effects (CPE) were not observed when cells were inoculated with filtered homogenate from heart of healthy salmon (negative controls; Fig. 1b).

Exposure of the HSMI inoculum to pH 3.0 abolished the development of CPE in inoculated GF-1 cultures. The cell cultures infected with virus inoculum exposed to pH 5 and 7.2 (standard culture medium conditions) developed CPE after 10 days of incubation. Treatment of inoculums at 70°C and 80°C resulted in loss of infectivity while inoculums treated at 60°C and lower temperatures were indistinguishable from positive controls showing distinct CPE in cell cultures post infection.

### The Genome Sequence of the Cell Culture Grown Virus is Identical to Piscine Reovirus (PRV)

This work was initiated prior to the sequence of PRV becoming available and a sequence independent approach for viral genome sequencing were applied on concentrated viral particles from cell culture. The sequences we obtained showed high similarity to viruses of the family of *Reoviridae* when blasted against the NCBI sequence database (blastn and blastx). One of the assembled contigs with a total length of 1097bp matched the lambda A-protein (major core/inner capsid protein) of an Avian orthoreovirus (E-value 6e-71). PCR primers (Table 1) based on this sequence were designed to screen cDNA from HSMI inoculated GF-1 cell cultures and tissues from Atlantic salmon challenged with cell culture grown virus and also used to test samples from salmon with an HSMI diagnosis from field outbreaks (not shown). A product of the expected size was amplified and sequenced, and resulted in identical sequence to the initial clone. Non-infected and PMCV or SAV-3 infected cell culture showed no amplification using these primers. This sequence was deposited in GenBank under accession number HM453201. When the full genome sequence of PRV became available [1] we found that our sequences covered approximately 27% of the genome published in GenBank under accession numbers GU994013-GU994022, including parts of segment 6 with 98,5–100% identity. To establish the character of the viral genome we performed nuclease digestion using Mung bean nuclease and RNase A under both low-salt (10 mM) and high-salt conditions (300 mM) on RNA isolated from the infected cell cultures. This showed double stranded RNA nature of this fragment, which fits into the characteristics of all viruses of the family *Reoviridae* (results not shown).

**Table 1:** 

Target gene	Forward primer	Reverse primer	Annealing temperature	Product size	Reference
β-actin	CCAGTCCTGCTCACTGAGGC	GGTCTCAAACATGATCTGGGTCA	59°C	75 bp	[54]
ASRV	CGTACCGCTTCTAACCAAGC	ACATGACGACGGACTCCAAT	58°C	215 bp	NCBI GenBank HM453201
CD4	GAGTACACCTGCGCTGTGGAAT	GGTTGACCTCCTGACCTACAAAGG	58°C	121 bp	[55]
CD8α	CACTGAGAGAGACGGAAGACG	TTCAAAAACCTGCCATAAAGC	56°C	174 bp	[55]
CD8β	CGCACACACCTCAACAACTC	ATTGATGCGCAGTGTGAAAG	58°C	153 bp	[55]
Granzyme A	GACATCATGCTGCTGAAGTTG	TGCCACAGGGACAGGTAACG	60°C	81 bp	NCBI GenBank BT046910
IFNγ	CTAAAGAAGGACAACCGCAG	CACCGTTAGAGGGAGAAATG	60°C	159 bp	NCBI GenBank AJ841811
IL-12β (p40)	CTGAATGAGGTGGACTGGTATG	ATCGTCCTGTTCCTCCG	59°C	108 bp	NCBI GenBank BT049114
IL-10	CGCTATGGACAGCATCCT	AAGTGGTTGTTCTGCGTT	59°C	81 bp	EF16028/EF16029
Mx	TGCAACCACAGAGGCTTTGAA	GGCTTGGTCAGGATGCCTAAT	60°C	78 bp	[54]

### ASRV Infected Cell Culture Preparations Results in HSMI Lesions

**Figure 2:**
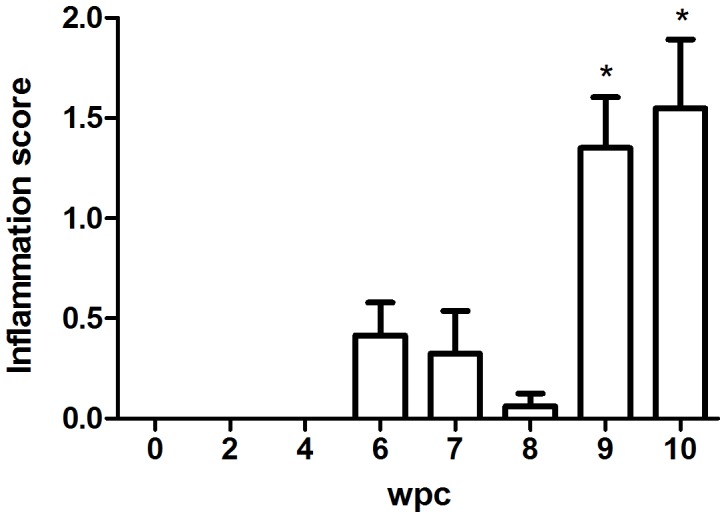
Histoscores at different time post challenge. Inflammation score following injection challenge examined at 0 to 10 weeks post challenge (wpc). Scores are 0 = no pathological changes, 1 = mild pathological changes, 2 = moderate pathological changes, 3 = severe pathological changes (for details, see text). Asterisk (*) indicates significant difference (p<0.05) to results from sampling at time 0 (all negative).

**Figure 3:**
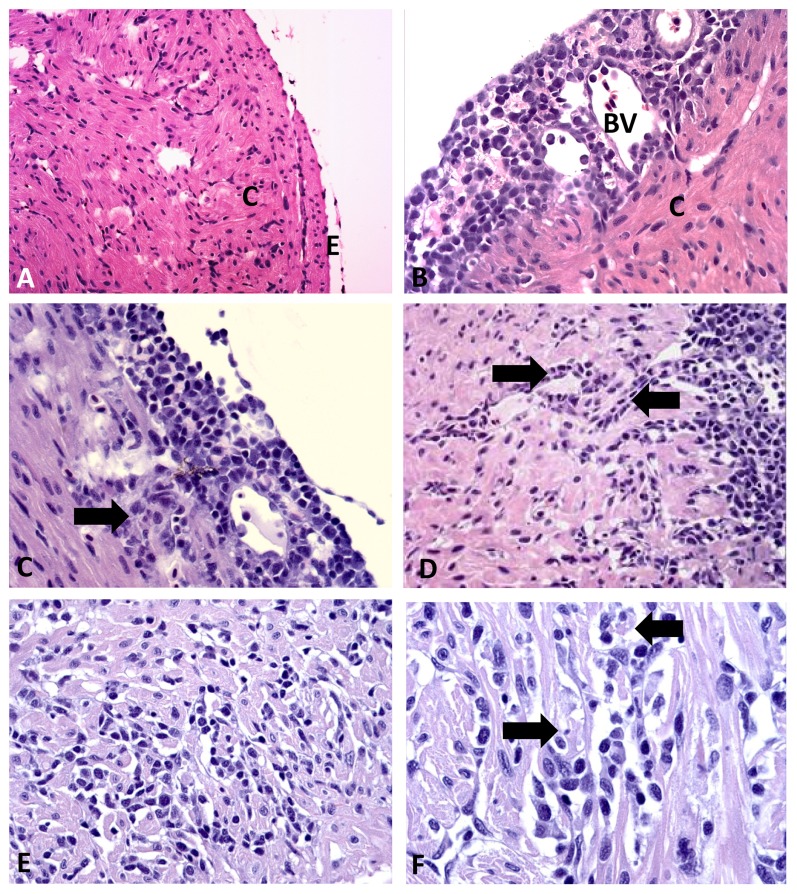
Histopathological changes in heart. Sequential histopathological of heart epicardium and the compactum of the ventricle at 4 to 10 weeks post challenge (WPC). All sections are counterstained with hematoxylin and eosin. A) 4 WPC with normal thin, epicardium (E) and normal compact layer (C) of heart ventricle; B) 7 WPC. Infiltration with lymphocytic cells in the epicardium located around small blood vessels (BV); C) 9 WPC. Epicardial infiltration extending into underlying compactum (arrow); D) 10 WPC. Epicardium (right) with infiltration of inflammatory cells along small vascular structures (arrows); E) 9 WPC and focal, infiltration of inflammatory cells in compactum of the heart ventricle; and F) 10 WPC showing myocardial necrosis (arrow) and moderate inflammation. x10 (A) and x40 (B–F), original magnification.

The next step was to document that first passage of HSMI tissue homogenate inoculated GF-1 cells (supernatant combined with a cell lysate from the same culture) caused HSMI lesions following experimental injection challenge of Atlantic salmon smolts and their cohabitants. The first cardiac lesions were seen at 6 weeks post challenge (wpc) in injection challenged fish (Fig. 2). Normal fish appeared without any inflammation of the epicardium (Fig. 3a) and infected fish had focal to diffuse lymphocytic infiltration in the epicardium. Inflammatory scores were similar at 7 wpc (Fig. 3b), lower by 8 wpc while at 9 and 10 wpc the inflammatory index increased with mean scores of 1.4 and 1.6, respectively (Figs. 2 and 3c,d). The main difference between early (6–7 weeks) and late time points (9–10) was that the inflammatory changes extended from the epicardium and into the compact layer of the ventricle, and “spreading” along small vascular structures (Fig. 3d). At late time points myocardial degeneration was also seen accompanied by infiltration of inflammatory cells (Figs. 3e–f). Histopathological changes were also seen in the red skeletal muscle, in 2 of 8 fish at 7 wpc, no fish at 8 wpc, 5 of 8 fish at 9 wpc and in 6 of 7 fish at 10wpc (not shown). All changes in heart and red skeletal muscle were typical of HSMI as described earlier [2], [3]. No mortality or external clinical signs were seen over the course of the challenge. This is consistent with what is found during early stages of natural outbreaks where morbidity can be high but the mortality is low [2], [3], [23].

### CD8^+^ T Cells Dominate in Inflamed Heart Tissue

**Figure 4:**
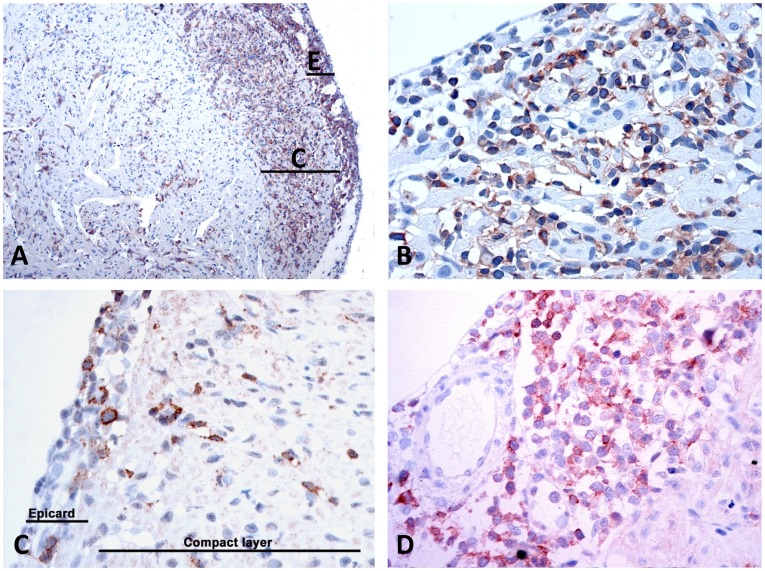
Immunohistochemistry for CD3 and CD8 positive cells. In situ detection of CD3- and CD8-positive T cells infiltrating the epicardium and underlying compact layer at 9 and 10 weeks post challenge. Positive cells are displayed with reddish/brownish color. All sections are counterstained with Mayer’s hematoxylin. A) CD3-positive cells infiltrating the epicardium, 9 WPC; B) CD3 positive cells with infiltration of the epicardium (top) and in the compact layer of the myocardium, 10 WPC; C) Overview of CD8-positive cells of the compact layer of the myocard (C) and epicardium (E), 9 WPC; D) Detail of CD8-positive cells infiltrating into the compact layer of the epicardium and into compact layer. Large proportion of lymphocytis cells are CD8-positive, 9 WPC. Original magnifications; x2.5 (A) and x40 (B–D).

We then went on to characterize the phenotype of the infiltrating cells at 4, 9 and 10 wpc using primary antibodies specific for T cells, CD3ε [19], and monoclonal antibodies specific for salmon CD8^+^ T cells [20]. Antibodies reactive with CD4^+^ cells are not available. At 4 wpc only a few single cells were found positive for CD3 or CD8, scattered throughout the heart tissue (not shown). This coincides with the histology results showing no lymphocytic infiltration at this time point. At 9 and 10 wpc there was a strong cytoplasmic staining for CD3 of the infiltrating cells in the epicardium (Fig. 4a) and with the inflammatory changes extending into the compact layer of the heart (Fig. 4b). Identical staining pattern was found for CD8 (Figs. 4c and d).

### Virus Load in Inflamed Heart Tissue Peaks Ahead of CD8^+^ T Cell Infiltration

**Figure 5:**
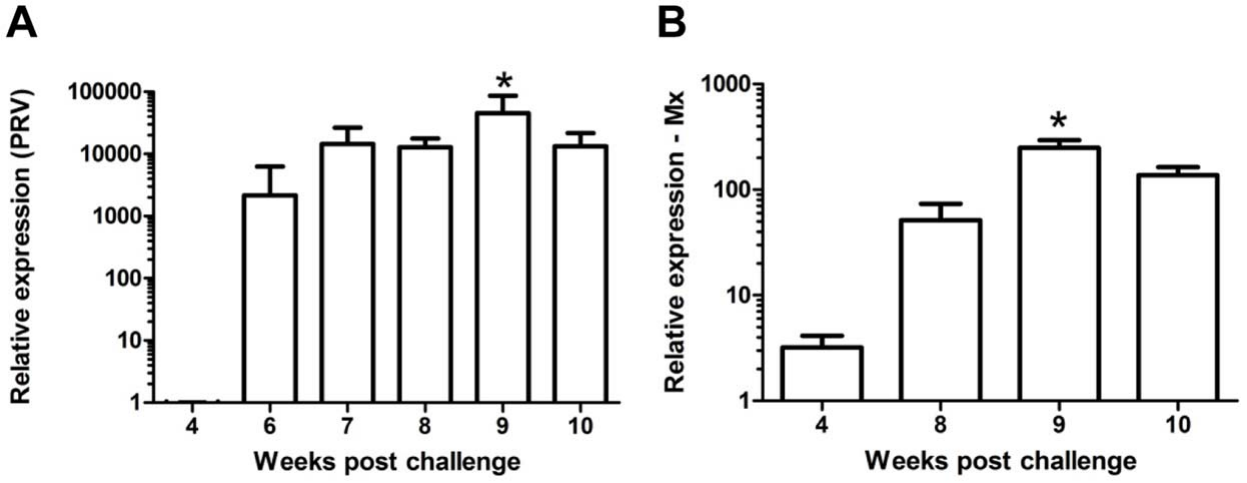
Expression of virus genome and Mx at different time post challenge. Relative expression of ASRV and Mx following injection challenge from 4 to 10 weeks post challenge (wpc). a) Relative replication level (log_10_) of ASRV in heart specimens was increased by 6 wpc and peaked at 9 wpc, which was the time point significantly different from all other samplings. b) Mx mRNA expression in heart tissue show peak expression at same time as virus replication. Different letters denote significant difference (p<0.05).

Our next approach was to document the virus load in heart of infected fish by real-time PCR at different times post challenge. Virus genome could not be detected from negative control fish (0 wpc) or from challenged fish at 2 wpc. By 4 wpc virus genome was detected in one of six fish tested (Cp value 33.3) and at 6 wpc there was a marked increase, one fish showing a more than 100 000-fold increase compared to the 4 wpc sample (Fig. 5a). Two of 6 fish were negative at 6 wpc. By 7 wpc and beyond ASRV genome was detected in all individuals tested (Fig. 5a). There was a peak at 9 wpc, with mean 45 000-fold increase relative to 4 wpc (P<0.05; Fig. 5a), reduced to 18 000-fold increase by 10 wpc. High virus load at 9 and 10 wpc also coincides with high inflammatory scores and myocyte necrosis at these time points (Figs. 2, 3e and 5a), but from 9 to 10 wpc virus load is on a decline while inflammatory scores are sustained at the same level at 9 and 10 wpc.

### The Infection Induces Expression of Antiviral Mx Protein

The type I interferon system is an important early line of defense against virus infections. Induction of type I interferons induces expression of the antiviral Mx protein, which is important in infections of several RNA viruses [24]–[27]. Mx mRNA expression levels in the inflamed heart samples studied peaked at 9 wpc in the injection challenged fish, representing a 78-fold change relative to the mean value at 4 wpc (P<0.001; Fig. 5b). Peak Mx expression coincided with the peak viral load. At 10 wpc Mx expression was still relatively high, though reduced compared to 9 wpc. When virus load was plotted against Mx in all fish individuals there was a moderate correlation between the two (r^2^ = 0.46).

### Gene Expression Profiling of the Cellular Immune Response Shows CD8 Polarization

**Figure 6:**
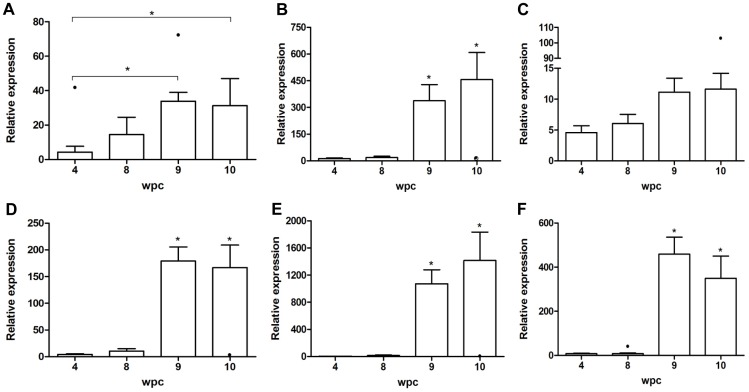
Relative expression of immune markers at different timepoints post challenge. Samples were collected at 4 to 10 wpc. A) and B) showing CD4 and CD8α expression, respectively. Asterisk (*) indicates significant difference (p<0.05) to 4 and 8 wpc or as indicated. Increased CD4 expression (A) was observed at the same time as for CD8α (B) while the peak expression level was much higher for CD8α, 450-fold versus close to 40-fold for CD4; C) IL12 expression was moderately increased while D) IFNγ and E) Granzyme A were markedly upregulated and peak expressions coincide with peak T cell levels. F) IL-10 was significantly upregulated at 9 and 10 wpc. Dot (•) indicates outlier values.

The next step included molecular profiling of the immune cells and associated cytokines by transcriptome analysis. In line with the infiltration of lymphocytic cells found by histology and immunohistochemistry against CD8^+^ and CD3^+^ cells, CD4 and CD8 (α-chain) mRNA expression in heart tissue from injected fish was low up to 8 wpc when a moderately increased expression of CD4 and CD8α was found (Fig. 6a and b, note different scales on y-axis). At 9 and 10 wpc CD4 and CD8α showed a marked increase, with a strong bias towards CD8α (Fig. 6b) with 18-fold change from 8 to 9 wpc (P<0.01) and a slight increase from 9 to 10 wpc. CD4 showed insignificant change from 8 to 9 wpc and did not increase any further by 10 wpc (Fig. 6a). Comparison of the inflammatory score and CD8α expression (as assessed by real-time PCR) showed a high degree of correlation (r^2^ = 0.81). The CD8 co-receptor on mammalian cells is expressed in two molecular isoforms with the αβ heterodimer being the major form present on circulating lymphocytes (thymocytes and mature T cells) [28], while the αα homodimer is exclusively present on subsets of NK cells and intraepithelial lymphocyte cells [29], [30]. Since both the CD8α- and β-chain are present in Atlantic salmon, we studied the expression of CD8β in parallel to CD8α (Data S1). The expression of the β-chain followed the same profile over time post challenge as was seen for CD8α (Fig. 6b), which indicates that the CD8αβ, characteristic of mammalian cytotoxic T cells, is the dominant isoform. The equal expression profile is also confirmed by a strong correlation when comparing relative expression of CD8α to CD8β in individual samples showed (r^2^ = 0.96).

The cytokine profile of third signal cytokines concomitant with CD8αβ up-regulation post viral infection has been described in common carp (*Cyprinus carpio* L.) [31]. IL-12 expression showed a moderate increase over time (4–10 wpc), however not significant between any of the samplings (Fig. 6c). In concert with what was observed for CD8, we found a similar expression profile for interferon γ (IFNγ) (17-fold change in mean expression from 8 to 9 wpc; P<0.01) and the effector molecule granzyme A (65-fold up-regulated from 8 to 9 wpc; P<0.01) (Fig. 6e). There was a good correlation between CD8 expression and granzyme A (r^2^ = 0.71). Collectively these findings show a consistent profile for CD8 (α- and β-chain), IFNγ and granzyme A and together point towards an important role for CD8^+^ cytotoxic T cells in the local immune reaction in the heart infected with ASRV. Interleukin 10 (IL-10) has been described as a cytokine with broad anti-inflammatory properties in mammals while the function in fish is not well understood. IL-10 expression was not differentially regulated up to 8 wpc while at 9 wpc there was a pronounced increase of expression, close to 55-fold increase (P<0.001), and still high at 10 wpc.

### Virus Spreads to Cohabitant Fish and Induces the Myocarditis and Myositis in Cohabitant Fish

Cohabitant fish were collected at 6, 8 and 10 weeks post challenge, the purpose being to document on virus spread in water and to explore if similar changes were observed in target organs. Inflammatory heart changes typical of HSMI were observed at 10 wpc (mean score of 1.80). Minor changes were observed at earlier samplings in a few individuals. The cohabitant fish also exhibited typical HSMI changes in red skeletal muscle, present in 1 out of 6 individuals at 6 wpc and 3 out of 8 at 10 wpc. Viral load were similar to what was found in fish challenged by injection and was detectable in all sampled fish at the three samplings, with low levels at 6 wpc and a significant increase from 6 to 8 and 10 wpc, and coincided with Mx expression. Also similar to the injection challenged was the gene expression profiling of the cellular immune response, which showed CD8 polarization with CD8α significantly increased at 10 wpc relative to both 6 and 8 wpc and a good correlation with profiles for IFNγ and the effector molecule granzyme A. These findings show that injected fish will shed virus which subsequently will infect cohabiting fish and lesions will develop in target organs.

## Discussion

In this study we show that Atlantic salmon reovirus can be passaged in cell culture and that experimental challenge of naïve Atlantic salmon with supernatant from cell culture passaged virus results in CD8 T cell myocarditis and red skeletal muscle myositis, typical of HSMI. Occurrence of inflammatory changes in the heart coincides with peak replication of virus. Virus is shed from infected fish and infects and replicates in cohabiting fish, where myocarditis and myositis is seen, further documenting the reproducibility and infectious nature of the disease.

In the study by Palacio and coworkers [1] where the full genome sequence of this reovirus was first presented, the authors showed that the virus was present also in fish without clinical signs and their findings indicated that the virus was widespread in Norwegian salmon farms. They also showed that the virus was found in wild Atlantic salmon [1]. Although this was at lower concentrations/prevalence than in diseased fish, these observations led them to suggest that PRV is the causative agent of HSMI. Further to this, a more recent study showed that PRV was also found in Atlantic salmon with cardiomyopathy syndrome [32], another infectious myocarditis of salmon caused by piscine myocarditis virus (PMCV) [33], and the authors described PRV as an opportunistic virus, which added doubt to the pathogenic traits or virulence profile of PRV.

Here, we have shown that fish challenged with in vitro passaged ASRV become infected and virus is detected as early as 4 weeks post challenge in heart tissue by real-time PCR. The virus load peaked concurrently with a marked increase in severity and prevalence of the myocarditis lesions. It has earlier been shown by *in situ* hybridization in heart tissue that PRV viral RNA localize to regions with typical HSMI lesions [1]. It should be added that the fish were negative for ASRV prior to infection and both challenge material and fish were negative for PMCV. These findings further support that reovirus infection is associated with HSMI. It should be noted that although ASRV can be passaged in cell culture, it has not been possible to obtain virus concentrations high enough to purify or quantify the virus or visualize the virus using electron microscopy. The method chosen for cloning and identification of virus from supernatant from GF-1 cultures inoculated with an HSMI homogenate is designed to isolate nucleic acids in solution that are protected against digestion after RNase and DNase treatment (i.e. covered by a protein shell). From this, total RNA was isolated, which means that both single-stranded and double-stranded RNA viruses potentially present in the original solution should be included. The procedure resulted in detection of sequences related to PRV only, with the exception of contaminating sequences with homology to fish cell related genes or non-virus related sequences with high concentration of tandem repeats. Since no other virus was found and the ASRV load is in accordance with histopathological characteristics typical of HSMI, the results obtained strengthen the hypothesis that ASRV infection is the cause of HSMI in the current study. Only one virus was also presented by Palacios et al [1] after pyrosequencing of RNA extracted from HSMI related heart tissue and serum. This shows that two separate attempts on cloning any agent with an RNA genome related to tissue or cell culture passaged preparations from two separate HSMI outbreaks both did not reveal any other agent but the reovirus. Still, the reovirus is observed in both farmed and wild fish not showing clinical signs of disease ([1], personal observation). A possible explanation might be the presence of low- or avirulent variants, the before mentioned possibility that the reovirus might be an opportunistic virus which cause disease when it can take advantage of a compromised immune system or that other factors combined with the presence of virus must be involved to result in disease in the field, which would be comparable to what is found for infectious pancreatic necrosis virus [34]. Since the virus has not been purified from tissue or cell culture there is a formal possibility that some other second agent could influence the disease progress. More work is needed to draw a firm conclusion.

The virus load in heart tissue showed good correlation with Mx expression (Fig. 5). In salmonids, several studies have shown induction of Mx expression as a result of virus infection [25]–[27]. Mx has an antiviral activity against a wide range of RNA viruses or virus producing RNA intermediates as part of their replication cycle [24] including fish viruses [25]. Peak Mx expression that coincides with peak virus load around week 9 and a concurrent decline by week 10 could indicate some involvement of Mx in controlling virus replication in addition to the T cell response also contributing to limit virus replication. Additional studies are needed to understand more of these interactions.

Myocarditis is per definition a condition characterized by inflammation of the heart muscle accompanied by degeneration and/or necrosis of adjacent cardiomyocytes [35]. The etiology of myocarditis in humans remains unknown in most cases but an association with a viral infection has attracted a lot of attention over the last years [36], [37]. Our findings show that HSMI fulfill the criteria of a classical myocarditis. When it comes to the immune mechanisms involved, teleost fish possess virus-specific cell-mediated cytotoxic activity and studies have shown that ginbuna crucian carp mount virus-specific cytotoxic responses where the phenotype and gene expression pattern are similar to those of cytotoxic T cells in mammals [38]. Antiviral cytotoxic cells have also been found in rainbow trout [39] while in salmon, no functional studies of virus-specific cytotoxic cells have been carried out since inbred strains of salmon are not available. The profile of the cells infiltrating the heart of HSMI fish is biased towards CD8^+^ over CD4^+^ cells. The concurrent increase of IFNγ expression also points towards a cell-mediated immune response in the heart [40] and further, the protease effector molecule granzyme A showed a pronounced increase in expression during the infection with very high expression levels in some fish (one fish having 3100 times upregulation at 10 wpc compared to 4 wpc), indicating a phenotypic profile of a cytotoxic T cell.

It should be added that mammalian NK cells can carry the same CD8α as in the CD8αβ co-receptor of cytotoxic T cells, but in αα homodimer isoform [29],[30]. NK cell equivalents producing granzymes have been found in teleosts [41]–[43], but whether these teleost NK-like cells can carry a CD8αα homodimer is not known. We found strong correlation between the expression of CD8α and CD8β in all samples. This makes it reasonable to conclude that a CD8αβ heterodimer characteristic of cytotoxic T cells is the dominant isoform present.

Our experiments show that HSMI leads to a remarkably high increase in mean expression of the cytokine IL-10. IL-10 has been cloned and characterized in several teleosts [44]–[49], and following viral infection it has been shown that IL-10 expression increases markedly in Atlantic salmon [50]. This seems to indicate that IL-10 is a cytokine involved in proinflammatory processes but whether teleost IL-10 functionally possesses the anti-inflammatory function equivalent to the mammalian counterpart remains to be confirmed. Since it has been shown that fish that go through a natural outbreak of HSMI recover and that the inflammatory changes subsides with time [23] it would be interesting to explore what role IL-10 plays in dampening the immune responses.

## Materials and Methods

### Culture Conditions and Physio-chemical Characterization of Infectious Agent

Heart tissue was collected from a clinical outbreak of HSMI and from non-related healthy salmon (controls). The heart tissue was homogenized followed by centrifugation at 4 000*× g* and 4°C for 20 minutes to remove cellular debris before processed through a 0.45 µm filter. The homogenate was subsequently inoculated onto GF-1 cell cultures [51]. The GF-1 cell line, derived from the fin tissue of orange-spotted grouper, *Epinephelus coioides*, was obtained from Schweitzer Biotech Company Ltd., Taiwan, PRC, and grown at 15°C in L-15 (Gibco) supplemented with 1% L-glutamine, 0.1% gentamicin sulphate (all from Sigma Aldrich) and 10% fetal bovine serum (Invitrogen). At 14 days post inoculation, supernatant and cell lysate were harvested and passaged.

The infectious agent was also subjected to various physiological conditions and subsequently inoculated on GF-1 cells cultured in 25 cm2 flasks to characterize the physiochemical properties of the agent. To determine the pH stability of the virus the pH of L-15 cell culture medium was adjusted to 5.0 and 3.0 by addition of 1.0 M HCl, and the pH adjusted L-15 solutions were sterile filtrated (0.20 µm). 350 µl test substances or control virus were incubated in 5 ml of the respective L-15 solutions in addition to normal L-15 (pH 7.2) for 4 hours at 15°C. After incubation, the pH was adjusted to 7.2 with 1.0 M NaOH before 2.5 ml of each solution were inoculated into two parallel 25 cm^2^ cell cultures in 5 ml growth medium. All flasks were incubated at 15°C for three weeks and microscopically examined twice per week. Thermal stability was tested by heating aliquots of the HSMI inoculum for 30 minutes at 25, 37, 45, 60, 70 and 80°C followed by immediate cooling by transfer to iced water. 170 µl of each aliquot were tested for residual infectivity by inoculation onto GF-1 cells. Control cells were inoculated with 170 µl of a non-treated aliquot. The cells were incubated for 3 weeks at 15°C.

### Cloning and Identification of the Virus Causing HSMI

A sequence independent approach for viral genome sequencing was performed for identification and partial cloning of the viral genome as described by Djikeng et al. with minor adjustments [52], [53]. In brief, GF-1 cultures were inoculated with a homogenate of organ material (heart) sampled in Nord-Trøndelag County in 2009 from Atlantic salmon (500 g size) with a histopathological diagnosis of HSMI. The supernatant was carefully collected after 21 days culture, centrifuged to remove cellular debris and processed through a 0.22 µm filter. The viral particles were concentrated by ultracentrifugation (Beckman Coulter Optima L-80 XP Ultracentrifuge). Approximately 30 ml were centrifuged in 10 ml aliquots at 100 000× g for 4 hours in a SW41 rotor (Beckman Coulter). Nearly all of the cell culture medium was carefully removed, before pellets containing virus were pooled and resuspended in a combination of the remaining and fresh cell culture medium, to a total of 200 µl. To enrich for viral particles and eliminate residual nucleic acid contaminants in the culture medium, 100 units of DNase I (Qiagen) and 10 µg/ml RNase A (Sigma-Aldrich) in RDD-buffer (Qiagen) was added to the viral resuspension and incubated at 37°C for 1 hour. RNA was isolated immediately after nuclease treatment using the QIAamp viral kit (Qiagen) according to the manufacturer’s instructions, however without using carrier RNA. Single stranded cDNA was synthesized using SuperScript®III reverse transcriptase kit (Invitrogen) and a random chimeric primer (1 µM) containing a fixed sequence of 20 nt and a random hexamer attached to its 3′-end (FR26RV-N primer: 5'-GCCGGAGCTCTGCAGATATCNNNNNN- 3'). RNA, dNTPs and the FR26RV-N primer were mixed and incubated at 95°C for 2.5 min prior to cDNA synthesis to avoid problems caused by a possible secondary structure of the RNA. The reaction mix was thereafter immediately transferred to ice before the enzyme mix was added and reverse transcription was performed as described in the kit protocol. Following heat inactivation of the enzyme mix, RNA complementary to the synthesized cDNA was removed by adding 2 units of E. coli RNase H (Invitrogen) and incubation at 37°C for 20 minutes. Double stranded cDNA was synthesized by Klenow reaction using a combination of the 2^nd^ strand buffer components of the Universal RiboClone® cDNA Synthesis System (Promega) and Exonuclease-free Klenow polymerase (Invitrogen). The reaction mix contained 10 µl first strand reaction, 20 µl second strand buffer, 2.5 µl acetylated BSA, 10 U Exo- Klenow polymerase, 0.2 µM random primers (FR26RV-N) and finally 15 µl nuclease-free water. The reaction was incubated at 37°C for 30 minutes, before inactivation of the enzyme by incubation at 70°C for 10 minutes. Finally a single primer PCR reaction using 5 µl of the double stranded cDNA, standard Taq polymerase (Invitrogen) and the FR20RV primer (5'-GCCGGAGCTCTGCAGATATC-3') was performed. The PCR products were separated by 1% agarose gel electrophoresis. Products in the size range 500–1000 bp of the resulting smear were excised from the gel and purified using QIAquick Gel Extraction Kit (Qiagen). PCR products were ligated into the pCR® 2.1 vector using TOPO® TA cloning® kit (Invitrogen) and transformed into competent OneShot® TOP10 bacterial cells (Invitrogen). After culture, 134 clones were picked and sequenced using a commercial service (GATC Biotech). Sequence reads were trimmed to remove vector sequence, amplicon primer sequence as well as low quality sequence, and assembled into contigs using the Contig Express program of Vector NTI advance 11.0 (Invitrogen). Sequences were first analyzed for repetitive elements using Tandem Repeats Finder (http://tandem.bu.edu/trf/trf.html) and only sequences without repeats were submitted to BLAST analysis (http://blast.ncbi.nlm.nih.gov/Blast.cgi). Putative sequence identity was determined based on BLAST similarity, and sequences matching cellular origin were discarded. A set of 17 remaining sequences was regarded as potential virus specific sequences. Standard PCR was performed based on primers designed from representatives of these sequences (Table 1) to screen cDNA from GF-1 cultures inoculated with virus originating from HSMI diseased fish, piscine myocarditis virus (PMCV) and salmonid alphavirus 3 (SAV-3), and on heart and kidney tissue from an HSMI diagnosed field outbreak.

### Challenge Experiment

The challenge experiment was approved by the Regional Committee for Medical and Health Research Ethics (in Norway) and was conducted in compliance with EU’s Welfare Act (86/609/EEC) and the Council of Europe’s Convention on Experimental Animals (ETS 123). Challenge of Atlantic salmon was performed using a cell culture 1^st^ passage supernatant of GF-1 inoculated cells combined with a cell lysate from the same culture. The organ material was collected from Atlantic salmon in 2003 with a histopathological diagnosis of HSMI. The samples were collected in Nordfjord, Sogn and Fjordane County on the west coast of Norway.

Since no sequences of a viral agent genome was available at the start of the study the 1^st^ passage preparation was chosen as this passage was found with more pronounced vacuolization (CPE) in the cell culture than later passages. The challenge material was kept frozen at −80°C until used. The cell cultures were originally inoculated with clarified heart tissue homogenate from a clinical outbreak of HSMI filtered through a 0.45 µm filter. A total of 80 Atlantic salmon (*Salmo salar* L.), strain Aqua Gen, were used in the experiment. The fish had been smoltified (prepared for sea transfer) according to standard procedures of the Industrial and aquatic laboratory (ILAB), Bergen, Norway. The fish were fed according to standard procedures, except for the day of challenge where the fish were kept off feeding. Water temperature was maintained at 12°C with a flow at 0.8 l/kg fish per minute. Fish were anesthetized and subsequently challenged by intramuscular (i.m.) injection of 0.1 ml on each side of the fish in the lateral muscle tissue beneath the dorsal fin (total 0.2 ml per fish). 20 untreated fish were subsequently marked by clipping the adipose fin and transferred to the same tank as the i.m. injected fish at the day of challenge. These fish served as cohabitants and were included to assess horizontal transfer of the infection. 10 control fish were sacrificed at the start of the experiment to document absence of any pathological changes and freedom of ASRV before challenge.

### Sampling of Tissue and Processing Procedures

Parallel samples for histology and real-time PCR studies were collected sequentially every second week post challenge (wpc) the first 6 weeks and subsequently every week up to 10 wpc for the injection challenged group (6 to 8 fish per sampling). At 6, 8, and 10 wpc parallel samples were also taken from the cohabitant fish (6 fish per time point). Heart and muscle tissues were submerged in 10% phosphate buffered formalin and kept at 4°C for at least 4 days until embedment in paraffin, performed according to standard procedures. Sectioning was performed at 4–5 µm and slides were counterstained with hematoxylin and eosin (H&E). For real-time PCR studies heart samples (10–30 mg) were placed in RNAlater and stored at 4 C for 1–3 days before long term storage on −20 C until RNA isolation. To assess the histological changes in the HSMI challenged fish, we used scoring by marking on a visual analog scale on the basis of the criteria given in Table 2.

**Table 2:** 

Inflammation score	Description of pathological changes
0	No pathological changes observed.
1	Mild pathological changes characterized by a limited number (countable) of mononuclear inflammatory cells infiltrating the epicardium, not extending into the compact layer of the ventricle. The infiltration of cells is multifocal to diffuse and can involve parts of or the entire epicardium available for assessment.
2	Moderate pathological changes consisting of high number (uncountable) of inflammatory cells in the epicardium and extending into the compact layer of the heart. The changes in the compact layer can be multifocal or diffuse and typically orient along small blood vessels. A few focal changes can also be seen in the spongious layer.
3	Severe pathological changes characterized by intense infiltration of inflammatory cells in the epicardium, extending into the compact layer, typically with a diffuse distribution pattern and involving the spongious layer in a multifocal pattern. Degeneration and or necrosis of muscle fibers are seen. Atrium can also be involved with inflammatory changes comparable to what is seen in the ventricle.

### Immunohistochemistry

To characterize the phenotype of the infiltrating cells in the inflamed myocardium, we used immunohistochemistry and antibodies against cellular markers as described below. These studies were performed on samples from time points 4, 9 and 10 wpc for injection challenged fish and at 10 wpc for the cohabitants. Unless otherwise stated, all the incubations described below were performed at room temperature. Commercially available anti-human CD3ε (DAKO) was used to stain T-cell like cells *in situ*. After demasking by autoclaving in 0.01 M citrate buffer pH 6.0 at 105°C for 15 minutes, endogenous peroxidase activity was inhibited by adding 3% H_2_O_2_ in methanol and non-specific binding was blocked by incubation in 5% bovine serum albumin (BSA) in Tris buffer. Primary antibody (anti-CD3) was incubated for 1 hour at 1∶100-dilution in 2.5% BSA in Tris buffer. The bound antibodies were detected using the EnVision™+ (DAKO; anti-rabbit) and 3-amino-9-ethyl-carbazole (AEC, DAKO) as chromagen. Sections were counterstained in Mayer’s hematoxylin and mounted in an aqueous mounting medium (Aquamount, BDH Laboratory).

A monoclonal antibody against salmon CD8α, Sasa CD8 F1-29 (kindly provided by Karsten Skjødt, Odense University, Odense, Denmark) was used to stain CD8-positive (CD8^+^) cells *in situ*. Demasking included autoclaving in 0.01 M citrate buffer pH 6.0 at 121°C for 15 minutes before inhibition of endogenous peroxydase activity using 0.05% phenylhydrazin in PBS for 40 minutes at 37°C. For blocking of non-specific binding and binding of the specific antibody with subsequent signal amplification, we combined the Avidin/biotin blocking kit (Vector labs) and Renaissance® TSA™ Biotin system reagents (Perkin Elmer, MA, USA). In short, initial blocking was done by 30 minute incubation in TNB-buffer (0.1 M TRIS-HCl, pH 7.5 and 0.15 M NaCl with kit Blocking reagent) with Avidin, 5∶1 (v/v). Primary anti-CD8 mab was incubated at 1∶50 dilution in TNB-buffer with Biotin (5∶1 (v/v)), at 4°C overnight. Biotinylated anti-mouse IgG1 1∶200 in TNB-buffer was used as secondary antibody. A signal amplification was done by incubation of Streptavidin-HRP reagent 1∶500-diluted in TNB-buffer followed by Biotinyl Tyramide reagent 1∶50 in Amplification diluents. A second incubation in Streptavidin-HRP reagent, 1∶100 in TNB-buffer build new biotin-streptavidin-HRP complexes around the antibodies, which then is detected using 3-amino-9-ethyl-carbazole (AEC staining kit, SIGMA) as chromagen. Sections were counterstained in hematoxylin and mounted in an aqueous mounting medium (Aquamount, BDH Laboratory).

### RNA Isolation

Total RNA was isolated using RNeasy® mini kit (QIAGEN) according to the manufacturer’s protocol. The procedure included on-column DNase treatment to minimize DNA contamination. The initial homogenization of each tissue were done in the kit lysis buffer using a mixer mill MM301 (Retsch® GmbH & Co) for 2 minutes at 20 Hz. The isolated RNA was quantified and purity analyzed using the OD260/280 ratio and a NanoDrop ND-1000 spectrophotometer (NanoDrop technologies).

### Real-time PCR

Production of cDNA from heart total RNA and subsequent real-time PCR were done using SuperScript™ III Platinum® Two-Step qRT-PCR Kit with SYBR® Green (Invitrogen) according to the manufacturer’s instructions. The kit uses high-temperature capability of SuperScript™ III Reverse Transcriptase (RT) in the cDNA-synthesis and combines automatic “hot-start” technology of Platinum® *Taq* DNA polymerase with integrated UDG carryover contamination prevention technology in the real-time PCR. In general, we transcribed 800 ng RNA into cDNA in a 40 µl reaction by mixing the RNA with the kit RT Enzyme and RT Reaction mixes, which includes both oligo (dT)_20_ and random hexamers for priming. After cDNA synthesis, the RNA template was digested using RNAse H to increase sensitivity in the real-time PCR.

Real-time PCR was run on the LightCycler 2.0 instrument (Roche). All reactions were performed in duplicates, each consisting of 2 µl template cDNA (undiluted when combined with CD4 and CD8 primers, diluted 1∶2 in water for the remaining reactions) in a 20 µl reaction. The reaction conditions were UDG-incubation at 50°C for 2 minutes, UDG inactivation, initial template denaturation and activation of the hot-start polymerase at 95°C for 2 minutes, followed by 40 cycles of 95°C for 15 seconds, primer annealing for 15 seconds (except for reactions using IFNγ- and Mx-primers) and extension at 60°C. Primer sequences and additional experimental conditions used in the real-time PCR are shown in Table 1. A melting curve analysis was performed to confirm formation of expected PCR products only. Products from all assays were additionally tested by agarose gel electrophoresis to confirm the correct size of the products. Data analyses were performed using the LightCycler software version 4.0. The crossing point (Cp) was determined by use of the maximum-second-derivative function on the LightCycler®Software. Cp values over 35 were considered negative after analyses of the melting curves showed no products with specific melting temperature. Analyses of gene expression were done with primer efficiency correction using external standards. Target gene expression was normalized against endogenous β-actin and expressed as relative to the sample with lowest expression being equal to 1. Too low RNA concentrations and quality were achieved from heart samples of the small fish in the beginning of the experimental challenge trial, which made the samples impossible to use in statistical expression analysis. Pooled samples of the RNA from 0 and 2 wpc showed expression with no significant difference to the 4wpc samples for any of the tested genes (not shown).

### Statistical Analysis

All statistical analyses were performed with the help of GraphPad Prism 5.0 (GraphPad Software Inc., USA). The level of significance was set at p<0.05.

## Supporting Information

Data S1 **Relative expression of CD8α and CD8β in infected and cohabitant fish.** The expression of CD8 α and CD8β correlated well over the challenge period both for injected (black color labels) and cohabitant (coh, gray labels) fish.
(TIF)
