# Supplementary figures and images for: Atlantic Salmon Reovirus Infection Causes a CD8 T Cell Myocarditis in Atlantic Salmon (Salmo salar L.)
Source: PLoS One. 2012 Jun 5;7(6):e37269. doi: 10.1371/journal.pone.0037269 (PMC3367920; doi:10.1371/journal.pone.0037269)

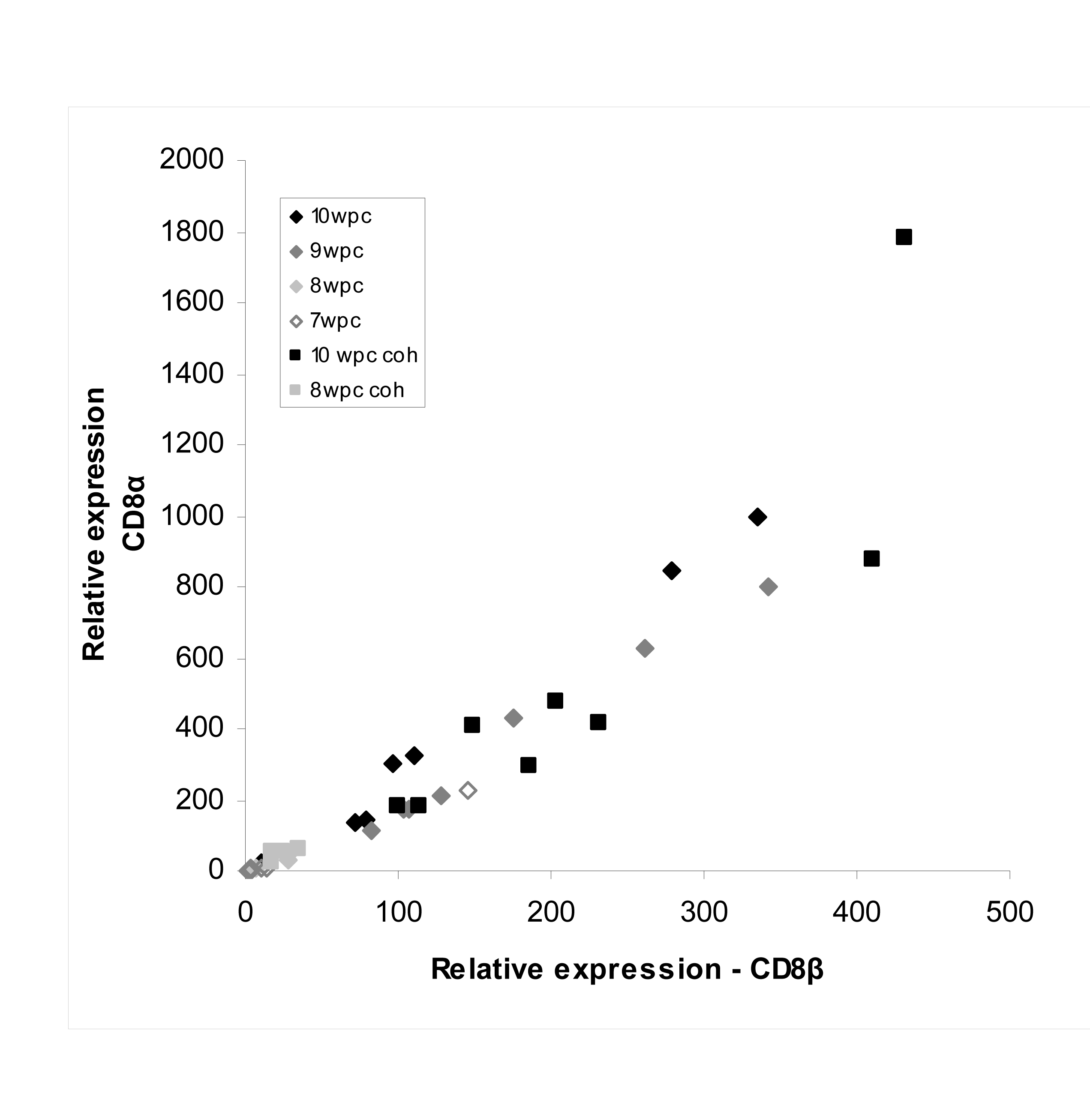

Supplement: Data S1 — Relative expression of CD8α and CD8β in infected and cohabitant fish. The expression of CD8 α and CD8β correlated well over the challenge period both for injected (black color labels) and cohabitant (coh, gray labels) fish. (TIF) [file pone.0037269.s001.tif]
